# Recommendation for post-exposure prophylaxis after potential exposure to herpes b virus in Germany

**DOI:** 10.1186/1745-6673-4-29

**Published:** 2009-11-26

**Authors:** Thomas Remé, Klaus Dieter Jentsch, Juliane Steinmann, Stephanie Kenner, Ulrich Straile, Eberhard Buse, Andreas Sauerbrei, Franz-Josef Kaup

**Affiliations:** 1 Institution for Statutory Accident Insurance and Prevention in the Health and Welfare Services, Department for Basic Sciences, Hamburg, Germany; 2 Department of Infection Pathology, Leipniz Institute for Primate Research, German Primate Centre, Goettingen, Germany; 3 Accident Insurance North Rhine-Westphalia, Department for Prevention, Duesseldorf, Germany; 4 Accident Insurance Baden-Wuerttemberg, Department for Prevention, Stuttgart, Germany; 5 Occupational health physician, Stuttgart, Germany; 6 Covance Laboratories GmbH, Muenster, Germany; 7 Institute of Virology and Antiviral Therapy, Friedrich-Schiller University, Jena, Germany

## Abstract

Although the risk of a herpes B virus (Cercopithecine herpes virus 1) infection is low, the clinical course of the infectious disease is generally unfavourable. A high safety standard can be achieved if people with professional contact to primates apply proper organisational, technical and personal safety precautions. The risk can be considerably reduced if animal keepers, laboratory assistants and scientists receive adequate information about the pathology of herpes B virus and are well trained in the necessary procedures and the precautions. For this reason, comprehensive and regular training, information and instruction must be provided to all primate workers and to laboratory workers who come into contact with potentially infectious material. After potential contamination, the risk for the affected worker must be assessed immediately and post-exposure chemoprophylaxis performed if necessary. This necessitates internal risk assessment. An interdisciplinary group of experts has developed an action plan for Germany.

## Introduction

Infection with the herpes B virus is rare, but nevertheless important, as transmission of the pathogen from macaques to human has led to sporadic deaths. Since 1930, over 43 infections with clinical symptoms have been diagnosed in different countries [1]. In Europe, no case of herpes B infection in man has been recorded. Herpes B virus infection is one of the most dangerous viral infections which can be transmitted from non-human primates to human. For this reason, safety precautions must be taken when humans are in contact with non-human primates. The risk can be considerably reduced by adequate management for primate workers and by paying attention to necessary safety standards. The risk is also reduced if the animals are carefully chosen during purchase, if animals are tested with the required serological pre-tests for herpes B virus and if the personnel are instructed about paying attention to the necessary safety precautions.

In humans, herpes B virus infection normally develops into encephalomyelitis, with an unfavourable prognosis [2,3]. As antiviral drugs have been produced (acyclovir, valacyclovir and famcyclovir, gancyclovir), new possibilities for post-exposure chemoprophylaxis exist [4]. Within 24 hours [4,5] after potential transmission of herpes B virus (e.g. bite by macaques, injuries, surface wounds or contact with body fluids), it must be decided whether antiviral prophylaxis should be started. This assessment ensures that the therapeutic window remains open. On the one hand, injuries are relatively common in animal keeping. On the other hand, herpes B virus transmission is extremely rare. Therefore, institutions with primate workers need professional risk assessment to bring about a quick and safe decision as to whether post-exposure chemoprophylaxis should be offered to an affected worker.

A first draft of this recommendation was presented at the symposium on herpes B infections on 13 November 2008 in the German Primate Centre in Goettingen and obtained explicit consent. Suggestions from the participants have been incorporated into the present version.

## Discussion

### Agent and primary host

Herpes B virus (normally known as B virus) is a DNA virus. The official nomenclature is "Cercopithecine herpesvirus 1". Another synonym is "Herpesvirus simiae". The virus is closely related to herpes simplex virus (human herpes virus 1, 2) of humans [5] in its structure and its virological and immunological characteristics.

B virus is an enzootic agent of Asian macaque monkeys (primary host). Natural infections, which are usually latent, have been described for monkeys, including *Macaca (M.) mulatta *(rhesus monkeys), *M. fascicularis *(long-tailed macaques), *M. radiata *(bonnet macaques), *M. fuscata *(Japanese macaques), *M. cyclopis *(rock macaques), *M. arctoides *(stump-tailed macaques) and *M. nemestrina *(pigtail macaques). Experimental or accidental infections, which mostly lead to death, have been reported for *Erythrocebus pata *(patas monkeys), *Colobus guereza *(guereza), *Cebus apella *(brown capuchin monkeys), *Callithrix jacchus *(common marmoset) and *Cercopthecus neglectus *(De Brazza's monkeys). Moreover, fatal clinical outcomes are known for African macaques (Barbary macaques, *M. sylvanus*). According to current knowledge, long-tailed macaques (cynomolgus, *M. fascicularis*) in the Mauritius Islands are free of the agent.

B virus may occur in natural reservoirs for macaques and in primate centres which keep macaques (zoos, animal keeping in research and industry, universities etc.). B virus is classified in risk group 3, according to the German guideline "TRBA 462" [6], corresponding to EG Guideline 2000/54/EG. The German Central Committee for Biological Safety (ZKBS) also classifies B virus in risk group 3 which corresponds at least to safety level S3 [7]. According to the Bio Hazard Guidelines, it is assumed that an organism of risk group 3 will be given special handling (see TRBA 462) during certain activities (e.g. storage of herpes B "without genetic engineering"). In such cases, the measures of protection level 3 must be ensured [6].

### Transmission route from primary hosts to humans

B virus is generally transmitted to humans percutaneously or transcutaneously by infected tissue or body fluids (saliva, blood) of macaques, especially after bites or scratches. Transmissions can also happen through wound or mucous contact with macaque saliva, injuries while handling animal enclosures accidents with cell cultures, needlesticks and with sharps during autopsy. Approximately half of the affected persons are animal keepers with direct contact to the animals. Another half is laboratory staff, e.g. while handling monkey cell cultures or blood components [8]. The last reported fatal disease in 1997 occurred through conjunctival infection [in Yerkes Regional Primate Centre, Lawrenceville] [9]. Only one transmission from human to human has been described so far (patient with contact dermatitis applied ointment on her husband's herpes blisters and then on her own skin) [10]. No cases of transmission to human have been identified in Europe, but it could happen at any time.

### Clinical picture

With the exception of Asian macaques, the primary hosts, non-human primates develop severe, mostly fatal clinical pictures with various changes in the organs, as well as severe vesicular exanthemas and pneumonia with giant cells and nuclear inclusion bodies, which are detectable by light microscopy [8].

After infections with B virus, human patients develop various symptoms, which normally develop within four weeks after exposure [Additional file 1]. Then the clinical picture develops further to severe ascending necrotising encephalomyelitis, which leads to death. The mortality rate is estimated to be more than 70% [3] [Additional file 1].

### Special diagnosis

If human exposure is suspected, the material must be collected from the suspect animal and tested in special institutions. The common detection methods are:

- Serological detection of B virus antibodies in serum via ELISA, IgG antibodies can only be detected quantitatively two to six weeks after the first infection with B viruses. For this reason, serological detection is only useful and necessary for retrospective analysis. However, this detection method is not necessary for considering and deciding about initiating therapeutic measures, as treatment must be started quickly if a patient may have been infected. Furthermore, it should be remembered that seronegative animals can be infected as well and may eliminate the virus before seroconversion, leading to the infection of other animals.

- Virological detection of B virus DNA through PCR in fluids from herpes blisters and CSF, otherwise post-mortem in spinal ganglia/trigeminal ganglia. DNA detection depends on the localisation and quality of the sampling and the clinical picture which the animal presents. The virus is persistent in ganglia. The location of the infected ganglia apparently depends on the site of entry [11,12]. During the short and sporadic viremic phases of B virus, viral DNA is readily detectable by PCR in the contents of herpes blisters, small ulcerations around the oral cavity and swab samples from the lips. It is more difficult to detect in serum. After death, PCR is suitable for DNA detection in ganglia (see above) and in samples from the tongue, liver, kidney, spleen, CNS or other tissues.

- Detection through culture of B virus in cell monolayers (Test material: smears from herpes blisters, CSF). Virus culture is the most reliable method but only possible in special laboratories under L4 conditions (see box).

A serum sample must be taken from the affected person for medical diagnosis after possible exposure. During international transport, guidelines must be considered when handling infectious material (UN 3373).

At the moment, there is no approved institution for handling human samples in Germany. For this reason, it would be desirable to establish a national reference laboratory [Additional file 2].

### Pre-exposure prophylaxis

In institutions keeping primates, the personnel must be regularly instructed about special risks and dangers of infection. The experience has been that this should be repeated every six months. Generally it must be assumed that, in doubtful cases, the macaque is excreting virus, so that a range of safety measures must be considered when handling the animals [Additional file 3].

### Post-exposure prophylaxis

Measures to be taken directly by affected persons (and first aid workers):

a) In the case of an injury to skin:

➢ Wound must be immediately and thoroughly cleaned for 15 minutes under running water and then disinfected (e.g. with 10% povidone iodine solution).

➢ Wound must be dressed superficially.

b) In the case of contamination of eye or oral cavity:

➢ Rinse under running water for approximately 15 minutes.

c) Further measures:

➢ Identification of the animal causing the infection.

➢ Documentation of the procedure.

➢ Consultation of the physician named in the accident kit [Additional file 3].

Measures to be taken by the employer:

a) Examination and testing must be performed as soon as possible after the accident by a suitable physician (see Pre-exposure prophylaxis, number 9). This includes virological and serological diagnosis and prophylactic antiviral therapy.

b) Testing and sampling (blood sample, saliva sample) by a veterinarian of the animal causing the infection.

c) Documentation of the course of the accident.

d) Notification to the responsible insurer of a suspected occupational disease, either by the involved doctor or by the employer.

Measures to be taken by the physician:

a) Immediately after the accident, it must be checked if the wound has been cleaned and disinfected or if the eye or oral cavity have been rinsed sufficiently. If necessary, repeat this and carry out wound care.

b) Clinical (neurological) examination of the affected persons must be carried out directly after the accident. Risk assessment must be done for the specification of a post-exposure chemoprophylaxis. Regular follow-ups must be performed at short intervals for approximately six weeks.

c) Serological diagnosis must be carried out directly after the accident (blank), as well as after three and six weeks. Later tests (e.g. three months after exposure) may sometimes be useful for patients receiving post exposure prophylaxis.

d) The patient must be informed about the clinical and prodromal symptoms and the agreement as to which measures should be taken in the case of occurrence (e.g. immediate consultation of the physician).

e) Information about chemoprophylaxis and, if required, initiation of treatment, as soon as possible after exposure. The recommended drug is valacyclovir (oral) three times daily, 1 g for 14 days. An alternative drug is acyclovir (oral) five times daily, 800 mg for 14 days [4]. As these drugs are not approved for prophylaxis of B virus infections (off-label use), it is necessary during use to consider the benefit risk assessment and to provide information as under study conditions.

f) Comprehensive medical documentation.

### Risk assessment in case of injuries from bites or scratches

The type of wound, the type of exposure, the depth of the wound and the localisation of the wound must be specified and a risk assessment must be carried out.

In the case of pathogen transmission through a wound on the head, the upper body or the neck, possible CNS changes may occur before other symptoms [Additional file 1] of B virus infection appear. Therefore, wounds on the head, neck and upper body should be assessed as high risk exposures. Studies on rabies virus, which progresses along the nerves from the periphery to the CNS, have shown that animal bites on the head or neck are more likely to lead to fatal disease than bites on the hands or fingers. As B virus progresses along the nerves into the CNS like rabies virus, the two viral infections may pose similar risks. Post-exposure chemoprophylaxis for potential exposures is recommended against B viruses if a wound has been suffered on the head, neck or upper body.

Deep wounds, especially those caused by bites, are more difficult to clean than superficial wounds. Therefore, decontamination of deep wounds may be relatively ineffective. For this reason, these wounds present a greater risk. Studies on rabies virus have shown that superficial wounds and scratches on the extremities less frequently lead to fatal disease than do deeper bites. Therefore, it is recommended [4] to perform post-exposure chemoprophylaxis in the case of deeper wounds with potential B virus transmission.

Superficial wounds and scratches can be cleaned easily and are therefore normally assessed as low risk exposures. [Additional files 4, 5 and 6].

### Risk assessment in the case of exposure to contaminated materials or objects

B virus exists in a latent form in the CNS of infected macaques and will be excreted intermittently from the mucous membrane of the infected animals. Therefore, injuries with needles and objects which may contain material from the CNS, the eyes, the eye lids or the mucous membranes, should be assessed as high risk exposure. There is no reliable information about the tenacity of B virus.

Although a case of viremia in a sick monkey has been described in the literature, it can be stated that viremia rarely occurs in clinically normal animals. Therefore, injuries with needles contaminated with the peripheral blood of clinically normal monkeys are assessed as low risk exposures. Sharp injuries have been associated with two cases of human B virus infection so far. One of the injuries occurred with a needle which was contaminated with the tissue from the eye of a monkey. The other injury was caused by a needle which had possibly been used for injecting a monkey [4,13] [Additional files 4, 5 and 6]

## Conclusion

There has been no case of herpes B disease in Germany in macaque keepers as of yet. However, injuries such as bites or needlesticks are frequent in animal workers and in the diagnostic laboratory. To avoid contamination with herpes B, pre-exposure preventive measures must be specified and implemented at the workplace. If contamination is suspected, post-exposure measures must also be precisely specified, so that the correct steps will be taken at once to ensure the necessary diagnosis and post-exposure chemoprophylaxis against herpes B viruses (herpes B-PEP).

These recommendations for the prevention and the post-exposure prophylaxis of herpes B have been developed in collaboration by occupational physicians, virologists, veterinarians and laboratory specialists. The initial draft has already been introduced and discussed during the symposium on "Herpes B virus infections" of the German Primate Centre (DPZ) in Goettingen on November 13, 2009. Suggestions made during this symposium have been incorporated into these recommendations.

This publication presents the first expert recommendations on pre- and post-exposure prevention after potential contamination with herpes B virus when working with macaques in Germany. The parties - the injured person, the employer and the (occupational) physician - now have a sustainable plan of action for the timing of the diagnostic and therapeutic procedure after a macaque bite and other similar accidents. This should provide them with confidence in their actions in the case of emergency.

## Competing interests

The authors declare that they have no competing interests.

## Authors' contributions

TR prepared and drafted the manuscript. All authors provided scientific information of their special fields and helped to draft the manuscript. All authors read and approved the manuscript.

## Supplementary Material

Additional file 1 **Symptoms suggestive of herpes B infection**. Symptoms suggestive of herpes B infection. Click here for file

Additional file 2 **Addresses for B virus diagnosis**. Addresses for B virus diagnosis in Germany. Click here for file

Additional file 3 **Safety measures for keeping macaques**. Safety measures for keeping macaques. Click here for file

Additional file 4 **Situations indicating post-exposure chemoprophylaxis**. Situations indicating post-exposure chemoprophylaxis. Click here for file

Additional file 5 **Situations with the possible indication of post-exposure chemoprophylaxis**. Situations with the possible indication of post-exposure chemoprophylaxis. Click here for file

Additional file 6 **Situations that do not warrant post-exposure chemoprophylaxis**. Situations that do not warrant post-exposure chemoprophylaxis. Click here for file

## References

[B1] Engel GA Jones-Engel L Schillaci MA Suaryana KG Putra A Fuentes A Henkel R Human exposure to herpes virus B-seropositive macaques, Bali, Indonesia Emerg Infect Dis 2002 8 8 789 795 12141963 10.3201/eid0808.010467 PMC3266706

[B2] Holmes GP Chapman LE Stewart JA Straus SE Hilliard JK Davenport DS Guidelines for the prevention and treatment of B-virus infections in exposed persons. The B virus Working Group Clin Infect Dis 1995 20 2 421 439 7742451 10.1093/clinids/20.2.421

[B3] Huff JL Barry PA B-virus (Cercopithecine herpes virus 1) infection in humans and macaques: potential for zoonotic disease Emerg Infect Dis 2003 9 2 246 250 12603998 10.3201/eid0902.020272 PMC2901951

[B4] Cohen JI Davenport DS Stewart JA Deitchman S Hilliard JK Chapman LE Recommendations for prevention of and therapy for exposure to B virus (cercopithecine herpes virus 1) Clin Infect Dis 2002 35 10 1191 1203 10.1086/344754 12410479

[B5] Weigler BJ Biology of B virus in macaque and human hosts: a review Clin Infect Dis 1992 14 2 555 567 1313312 10.1093/clinids/14.2.555

[B6] TRBA 462 Einstufung von Viren in Risikogruppen. Bekanntmachung des BMA vom 01. November 1998 Bundesarbeitsblatt 1998 12 41 43

[B7] Liste risikobewerteter Spender- und Empfaengerorganismen für gentechnische Arbeiten - Bekanntmachung nach § 5 Absatz 6 Gentechnik-Sicherheitsverordnung in der Fassung der Bekanntmachung vom 14.3.1995 (BGB1. I S. 297) Bundesgesundheitsblatt Gesundheitsforschung Gesundheitsschutz 2001 44 3 246 304 10.1007/s001030050440

[B8] Diagnose und Praevention der Herpes B-Virusinfektion Schwibbe M, Goettingen Eine Bestandsaufnahme DPZ-Symposium 03.12.1998 1999

[B9] Fatal Cercopithecine herpesvirus 1 (B virus) infection following a mucocutaneous exposure and interim recommendations for worker protections MMWR Morb Mortal Wkly Rep 1998 47 49 1073 6 9879633

[B10] B-virus infection in humans--Pensacola, Florida MMWR Morb Mortal Wkly Rep 1987 36 19 289 6 3033462

[B11] Oya C Ochiai Y Taniuchi Y Takano T Fujima A Ueda F Hondo R Yoshikawa Y Prevalence of B-virus genome in the trigeminal ganglia of seropositive cynomolgus macaques Lab Anim 2008 42 1 99 103 10.1258/la.2007.006031 18348771

[B12] Jentsch KD Kaup FJ Die Herpes B-Virusinfektion von Makaken und Zoonosegefahren für den Menschen Manuscript in preparation 2009

[B13] Artenstein AW Hicks CB Goodwin BS Jr Hilliard JK Human infection with B virus following a needlestick injury Rev Infect Dis 1991 13 2 288 291 1645881 10.1093/clinids/13.2.288

