# Supplementary material for: Recommendation for post-exposure prophylaxis after potential exposure to herpes b virus in Germany
Source: J Occup Med Toxicol. 2009 Nov 26;4:29. doi: 10.1186/1745-6673-4-29 (PMC2789725; doi:10.1186/1745-6673-4-29)
Supplement: Additional file 1 — Symptoms suggestive of herpes B infection. Symptoms suggestive of herpes B infection. [file 1745-6673-4-29-S1.doc]

**Additional file 1: Symptoms suggestive of herpes B infection**

| - Vesicular lesions of the skin at the site of injury (herpes blisters not obligatory), lymph node swelling is possible |
| --- |
| - Local itching, pain and numbness (local neurological symptoms) |
| - Influenza-like pain in the extremities |
| - Fever and chills |
| - Headache for more than 24 hours |
| - Fatigue for no evident reason |
| - Movement disorders |
| - Dyspnoea (shortness of breath) |
