# Supplementary material for: Recommendation for post-exposure prophylaxis after potential exposure to herpes b virus in Germany
Source: J Occup Med Toxicol. 2009 Nov 26;4:29. doi: 10.1186/1745-6673-4-29 (PMC2789725; doi:10.1186/1745-6673-4-29)
Supplement: Additional file 2 — Addresses for B virus diagnosis. Addresses for B virus diagnosis in Germany. [file 1745-6673-4-29-S2.doc]

**Additional file 2: Addresses for B virus diagnosis**

| If required, non-human samples can be examined in the German Primate Centre with ELISA and PCR. Information about herpes B is available there, too.  German Primate Centre (GCP)  Department of Infectious Pathology  Kellnerweg 4  37077 Göttingen  Germany  Professor Franz-Josef Kaup: 0551-3851-241 ([fkaup@dpz.gwdg.de](mailto:fkaup@dpz.gwdg.de))  Klaus Dieter Jentsch MD: 0551-3851-157 ([jentsch@dpz.eu](mailto:jentsch@dpz.eu))  Information in the internet:  DPZ/Infektionsforschung/Infektionspathologie/Herpes-B Virusinfektion  Besides non-human samples, samples for human diagnosis can be examined in the following approved institutions:  B Virus Research  and Resource Laboratory  Georgia State University, Atlanta, USA  Health Protection Agency, London, England  Viral Zoonosis Unit (VZU)  VRL-Laboratories  7540 Louis Pasteur  San Antonio, Texas, USA  Detailed information about this can be collected from the Guidelines of the American B Virus Group (4).  Other rapidly available information:  [http://www.haz-map.com](http://www.haz-map.com/) (then: Index of Diseases: Infectious Diseases: B-virus meningoencephalitis)  Recommendations on the samples to be collected and other information is available in the National B Virus Resource Centre of the Viral Immunology Centre of Georgia State University, USA (Internet Google search, key word: National B Virus Resource Centre). |
| --- |
