# Supplementary material for: Recommendation for post-exposure prophylaxis after potential exposure to herpes b virus in Germany
Source: J Occup Med Toxicol. 2009 Nov 26;4:29. doi: 10.1186/1745-6673-4-29 (PMC2789725; doi:10.1186/1745-6673-4-29)
Supplement: Additional file 3 — Safety measures for keeping macaques. Safety measures for keeping macaques. [file 1745-6673-4-29-S3.doc]

**Additional file 3: Safety measures for keeping macaques**

| 1. The aim must be to have a B virus free monkey colony. |
| --- |
| 1. Regular veterinary medical examination and serological testing of the animals |
| 1. For positively tested animals, suitable measures must be specified in individual cases (quarantine, separation and, if necessary, euthanasia). |
| 1. Avoid direct contact with animals that are not anaesthetized. |
| 1. Contact with the animal only for specially trained workers. |
| 1. Generally wear equipment for protection (protected clothing, suitable safety gloves, oral protection, safety glasses). |
| 1. Training and instruction of the workers as to the risks and the corresponding safety measures before starting the activity and subsequent regular repetitions (once yearly) and documentation. |
| 1. Control of compliance with safety measures. In the case of non-compliance: warning and repeating training, instruction and documentation. |
| 1. Agreement with a physician with experience in diagnosis and chemoprophylaxis after B virus contamination. |
| 1. Availability of a quick and readily accessible accident kit at a place where it is possible to wash the hands with suitable and sufficient wound disinfectant (10 % povidone iodine solution). An eye bath, dressing and the address or telephone number of the responsible physician should be available. |
| 1. Special training for the first aid workers according to Accident Prevention Guideline BGV/GUV-V A 1. |
