# Supplementary material for: Recommendation for post-exposure prophylaxis after potential exposure to herpes b virus in Germany
Source: J Occup Med Toxicol. 2009 Nov 26;4:29. doi: 10.1186/1745-6673-4-29 (PMC2789725; doi:10.1186/1745-6673-4-29)
Supplement: Additional file 4 — Situations indicating post-exposure chemoprophylaxis. Situations indicating post-exposure chemoprophylaxis. [file 1745-6673-4-29-S4.doc]

**Additional file 4: Situations indicating post-exposure chemoprophylaxis**

| 1. Skin exposure (with loss of skin integrity) or exposure of mucosa (with or without  signs of injury) to high risk source. The term high risk source means a macaque which is known as virus carrier or which has lesions compatible with a B virus infection.   - After bites or scratches by macaques or - Contact with   - conjunctival, oral or genital secretions,   - nerve tissue or materials which were contaminated by macaques (e.g. cages or equipment). |
| --- |
| 2. Insufficiently cleaned and disinfected skin after exposure – also on distal body  parts – with injury of the skin integrity or mucosa exposure (with or without signs  of injury) |
| 3. “Laceration” (cut, tear, flesh wounds) on the head, neck or upper body |
| 4. Deep bite wounds |
| 5. Sharps injuries from needles which are contaminated with tissue or fluids from the  CNS, suspected herpes B virus skin lesions, eye lids or mucosa |
| 6. Sharps injuries or cuts after exposure to   - Objects which are contaminated from secretions of macaques from herpes lesions of the mouth or genitals or nerve tissue or - Objects which are known to be contaminated with herpes B viruses |

b
