# Supplementary material for: Recommendation for post-exposure prophylaxis after potential exposure to herpes b virus in Germany
Source: J Occup Med Toxicol. 2009 Nov 26;4:29. doi: 10.1186/1745-6673-4-29 (PMC2789725; doi:10.1186/1745-6673-4-29)
Supplement: Additional file 5 — Situations with the possible indication of post-exposure chemoprophylaxis. Situations with the possible indication of post-exposure chemoprophylaxis. [file 1745-6673-4-29-S5.doc]

**Additional file 5: Situations with the possible indication of post-exposure chemoprophylaxis**

| 1. Mucocutaneous exposure which has been properly cleaned and disinfected |
| --- |
| 2 . Laceration with loss of skin integrity which has been properly cleaned  and disinfected |
| 3. Sharps injuries from needles contaminated with blood of a clinically abnormal macaques |
| 4. Sharps injuries and cuts after exposure to  (a) objects contaminated with macaque body fluids which do not originate from herpes B virus lesions or  (b) material from potentially infected cell cultures |
