# Supplementary material for: Recommendation for post-exposure prophylaxis after potential exposure to herpes b virus in Germany
Source: J Occup Med Toxicol. 2009 Nov 26;4:29. doi: 10.1186/1745-6673-4-29 (PMC2789725; doi:10.1186/1745-6673-4-29)
Supplement: Additional file 6 — Situations that do not warrant post-exposure chemoprophylaxis. Situations that do not warrant post-exposure chemoprophylaxis. [file 1745-6673-4-29-S6.doc]

**Additional file 6: Situations that do not warrant post-exposure chemoprophylaxis**

| 1. Skin exposure without injury of skin integrity |
| --- |
| 2. Exposures to other non-human primate species (cf. page 2, Chapter “Pathogen and  Primary Host”) |
